# Is global citizenship a priority in occupational therapy education? The perceptions of Australian occupational therapy educators

**DOI:** 10.1111/1440-1630.70083

**Published:** 2026-03-17

**Authors:** Shinead Borkovic, Tracy Fortune, Betty Leask

**Affiliations:** ^1^ Discipline of Occupational Therapy, School of Allied Health, Human Services and Sport La Trobe University Bundoora Victoria Australia; ^2^ La Trobe University Bundoora Victoria Australia

**Keywords:** cultural responsiveness, cultural safety, education, global citizenship, occupational therapy, phenomenography, political thinking, professional identity, social responsibility

## Abstract

**Introduction:**

In an increasingly interconnected world, there is a pressing need for occupational therapy students to graduate as culturally responsive and socially responsible global citizens. However, little is known about the extent in which global citizenship is understood by occupational therapy educators and if it is embedded in Australian occupational therapy education.

**Methods:**

An interpretive, phenomenographic approach was adopted, to identify the qualitatively different ways that occupational therapy educators understand global citizenship in occupational therapy education and its perceived importance in preparing graduates for professional practice. Twenty Australian educators were interviewed. Analysis of interview transcriptions generated four distinct ‘categories of description’ grounded in participants' awareness and experience of global citizenship and its relationship to internationalisation of the curriculum in occupational therapy education.

**Consumer and Community Involvement:**

No consumer/community were involved in this research.

**Findings:**

The four categories revealed global citizenship and internationalisation of the curriculum was perceived by participants as either *institutionally imposed and irrelevant*, *locally mandated and important*, *values driven and desirable*, or *professionally essential and aspirational*. While some participants described it as lacking relevance to occupational therapy education, others felt that it was essential to preparing students for professional practice and therefore should be in curriculum.

**Conclusion:**

Global citizenship in occupational therapy education was understood in various ways. Becoming a global citizen was considered by most participants as necessary for developing a strong professional identity. Further research related to student perceptions of global citizenship and how this contributes to the development of professional identity is called for.

## PLAIN LANGUAGE SUMMARY

Preparing occupational therapists as global citizens is required because the settings in which occupational therapists work are increasingly impacted by social, cultural, and political issues. Global citizenship is a commitment to addressing world issues, power, and injustice through actions, behaviours, and developing attributes associated with political thinking. The World Federation of Occupational Therapists has called for a stronger focus on global citizenship in occupational therapy education; however, little is known about global citizenship in occupational therapy education in Australia. In this study, 20 occupational therapy academics in Australia were interviewed, to identify the different ways global citizenship was understood and if it was embedded in curriculum. Analysis of the interview data revealed differences in perspectives with some educators stating global citizenship lacked relevance, while others felt it was either locally important, desirable to include in curriculum or essential for preparing future work‐ready graduates with a strong professional identity. Future research on global citizenship from a student perspective is recommended.

**Key Points for Occupational Therapy**

- Preparing occupational therapy graduates as global citizens is essential.
- Becoming a global citizen requires skills in political awareness, social responsibility, cultural humility and cultural responsiveness.
- Global citizenship should be embedded in occupational therapy curriculum to prepare students with a strong professional identity.

## 1 INTRODUCTION

Australia is a diverse, multicultural country. Aboriginal and Torres Strait Islander Peoples, settler‐colonialists, and high rates of migration contribute to the diversity within the Australian context through race, language, ethnicity, socio‐economic status, education levels, and different cultural and political beliefs. According to the Australian Bureau of Statistics (ABS, 2021a), 29.8% of the total population are born overseas. In June 2024, 445,600 people migrated to Australia during the previous 12 months, with the largest group identified as international students (ABS, 2024). According to the last census release in 2021, Aboriginal and Torres Strait Islander People make up 3.2% of the population (*n* = 812, 728) and speak 167 different languages highlighting diversity within cultures (ABS, 2021b). The breadth of cultural diversity within Australia points to the need to better understand the extent to which occupational therapy graduates are prepared with global citizenship capabilities for professional practice with diverse populations.

Preparing students for culturally diverse practice is a core responsibility of occupational therapy educators (Gray et al., 2024). According to the World Federation of Occupational Therapists (WFOT, 2021), graduates should demonstrate social responsibility and global citizenship, which requires curricula that promote transformative learning (Fortune et al., 2019; Malfitano & Lopes, 2018). Global citizenship entails ethical action to address global issues, power imbalances, and injustice (Elliot, 2015; Salter & Halbert, 2017). Andreotti (2006) frames critical global citizenship as empowering ‘individuals to reflect critically on the legacies and processes of their cultures, to imagine different futures and to take responsibility for [their] decisions and actions’ (p. 48). Social responsibility involves responding to global occupational challenges rooted in social, political, economic, and environmental inequities (Gutman, 2025; Lopes et al., 2025). Integrating these concepts into occupational therapy education aligns with WFOT's (2021) call to prepare graduates, in their local context, as socially responsible, work‐ready global citizens.

The internationalisation of the curriculum framework integrates international, intercultural, and global dimensions into curriculum content, outcomes, assessments, teaching methods, and student support (Leask, 2010, 2012). It supports culturally inclusive teaching, acknowledges power and oppression, fosters critical reflexivity, connects global and local practices, promotes global citizenship and social justice, and empowers students to challenge inequality (De Vita, 2007; Leask, 2012; Vishwanath & Mummery, 2018). However, little is known about how educators approach the task of incorporating global citizenship in occupational therapy education and whether the framework is utilised. This study aimed to explore educators' understanding of global citizenship and its role in preparing students for culturally diverse practice.

## 2 METHODS

### 2.1 Ethics

Ethics approval was granted by the La Trobe University Human Ethics Research Committee (HEC18267).

### 2.2 Study design

Phenomenography explores the qualitatively different ways individuals experience, conceptualise, and understand aspects of the world. The variations of participants' experiences were identified through line‐by‐line readings of interview transcripts and categorised in two ways. First, the data were categorised by similarity. Second, the categories were hierarchically ordered to reveal progressively advanced conceptualisations and/or experiences of global citizenship and the internationalisation of curriculum framework in occupational therapy education within an ‘outcome space’ (Akerlind, 2017; Marton, 1986). The outcome space highlights the variations between categories and includes specific details about where global citizenship was placed in the curricula, what the main focus of awareness was in participants' perceptions/experiences, the drivers of their perceptions, and examples of teaching practices that contribute to the variations between categories. This approach has been widely adopted in other education‐focused studies (Akerlind, 2017; Kilinc & Aydin, 2013; Tight, 2015).

### 2.3 Participants and recruitment

Participants were recruited through a website search of Australian university occupational therapy programs. Course Coordinators or Heads of School from 20 programs received email invitations including the participant information statement and consent form. Snowball and purposive sampling (Liamputtong & Ezzy, 2013) were used to reach eligible participants, including those in relevant non‐university educational roles. Eligible participants returned signed consent forms via email to the primary author and an online interview was scheduled. Participants were provided with a follow‐up email that included the study purpose, a definition of global citizenship (Andreotti, 2006), and three preparatory questions to reflect on prior to the interview. The questions were (1) the diversity of the Australian population has increased in the past decade. Have you noticed this diversity being reflected in the student population? And/or client populations in professional practice? (2) How do you see this impacting on the education we need to provide for occupational therapy students? (3) What strategies do you find best work best to do this?

### 2.4 Data collection

Twenty educators participated in 30‐ to 45‐min Zoom recorded semi‐structured interviews with the first author. Open‐ended questions were asked (see Table 1) enabling choice in response to the question (Liamputtong & Ezzy, 2013; Walsh, 2000). Data were collected from January to May 2019. Audio recordings were transcribed verbatim and analysed.

**TABLE 1:** Phenomenographic semi‐structured interview guide.

Questions	
Internationalisation of curriculum focus	Can you tell me a little bit about what you do to accommodate diversity in the classroom?
	What approaches do you use to educate students about culturally diverse perspectives in occupational therapy and why?
	Can you tell me about your international partnerships and the international placements in your occupational therapy program?
	In your opinion, is being globally aware of occupational therapy practice important? Can you describe how your occupational therapy curriculum is designed to support this?
	What are your beliefs about students learning from each other (such as peer review, sharing experiences, or collaborative work) both locally and internationally?
Internationalisation at home focus	Can you tell me about international virtual learning opportunities for students with other universities? If you do not offer this, do you view this as an important learning opportunity and experience for students and why?
Global citizenship focus	In your view, what would an ‘ideal’ global graduate look like in occupational therapy?
	How do you include critical reflection learning activities in your curriculum, and do you feel this is an important skill for students to practice? Why?
	Can you tell me about how you educate students on human rights and ethics?
	What (if any) aspects of your occupational therapy curriculum do you think prepare students with the capabilities associated with global citizenship?
	What do you think are the barriers/challenges to embedding global citizenship across all year levels of your curriculum?
Other	From the examples you shared with me today, do you feel you are guided by your institution, the discipline, or your own personal/professional values?
	What do you understand about the term global citizenship and internationalisation? Internationalisation of the curriculum? Do you see them as having any relevance to the conversation we just had?

### 2.5 Data analysis

**FIGURE 1:**
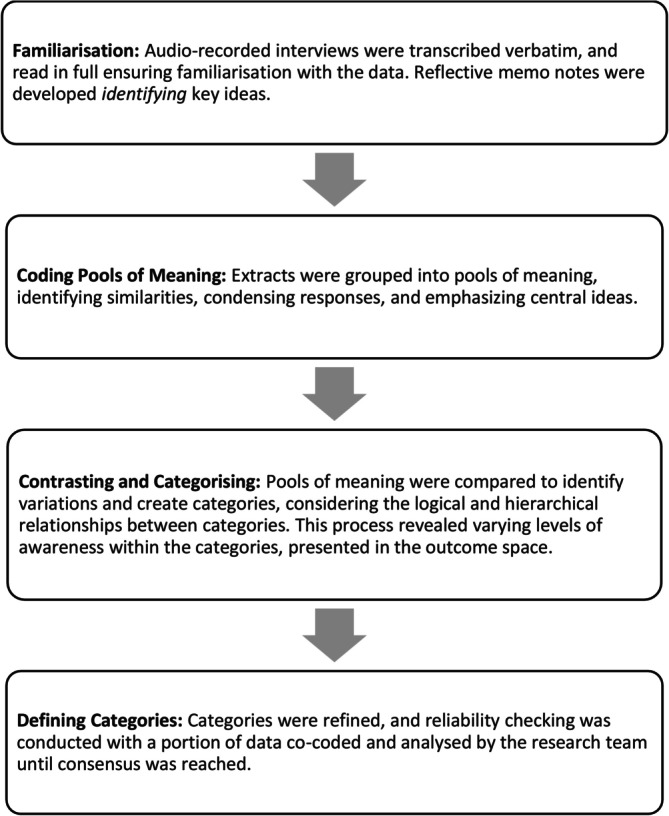
Process used to conduct phenomenographic analysis (Dahlgren & Fallsberg, 1991; Marton, 1992).

Data were analysed using methods by Dahlgren and Fallsberg (1991) and Marton (1992) with interview transcripts categorised by similarity and then positioned in a hierarchy to determine the difference/variation between categories. This process identified four qualitatively distinct categories reflecting how participants understood and/or experienced global citizenship in occupational therapy education with concepts increasing in complexity from the first category to the fourth. Responses from participants often spanned multiple categories, depending on participants' levels of awareness when responding to questions asked in the interview. The analysis process is outlined in Figure 1.

Phenomenographic analysis examines both referential and structural aspects of a phenomenon. In this study, internationalisation and global citizenship were determined by the authors as referential, representing participants varied perceptions of their importance. Structural aspects—teaching practices—were guided by Leask's (2010, 2012) formal (planned), informal (extracurricular activities), and hidden curricula (classroom discussions), and influenced by local, national, and global dimensions of the framework, along with Andreotti's (2006) critical and soft approaches to global citizenship education. A soft approach to global citizenship focuses on the moral responsibility of individuals to humanity. Positions of power are seen as a result of history, education, harder working communities, and better use of resources such as technology. From this viewpoint, problems of social and political injustice are due to a lack of culture, education, resources, and development. In contrast, a critical approach to global citizenship education requires self‐reflection on responsibility and action about how one contributes to worldwide social and political injustice. From a critical standpoint, injustice is derived from power relations, assumptions, and attitudes that continue to create and maintain exploitation and disempowerment (Andreotti, 2006). After 20 interviews, no new information emerged during preliminary analysis and data collection ceased (O'Reilly & Parker, 2013).

## 3 POSITIONALITY

All authors are Australian‐born women of Anglo‐European origin with diverse cultural, religious, and heritage backgrounds. Authors 1 and 2 are occupational therapy academics with strong interests in education‐focused research related to global citizenship and culture, while the third author is a professor of education with international expertise in curriculum internationalisation. All authors engaged in regular reflexive discussions and maintained an audit trail to ensure the research's credibility and dependability (Liamputtong & Ezzy, 2013).

## 4 FINDINGS

### 4.1 Participant characteristics

Demographically, participants varied in academic experience and brought diverse practice backgrounds, including mental health, paediatrics, and community. The geographic diversity of participants across Australia was a key strength, providing a broad range of perspectives. Participants were assigned unique codes to protect individuals' anonymity.

### 4.2 Categories of description

Four distinct categories were generated representing the qualitatively different ways that participants understood global citizenship and internationalisation of the curriculum in occupational therapy education, highlighting an increasingly advanced understanding from Categories 1 to 4.

#### 4.2.1 Category 1: Institutionally imposed and irrelevant

Internationalisation of the curriculum and global citizenship were conceptualised by some participants as imposed concepts by institutions and an ‘add on’ to curricula with little value or relevance to existing discipline specific curriculum. As one participant stated, ‘it becomes a checkbox activity of … we need to meet these outcomes … we need to have a case study … an international experience … but … is that international experience adding any quality to the students' learning?’ (P5). Participants associated internationalisation of the curriculum with a commercially driven agenda by institutions focused primarily on profit through international student recruitment, offshore programs, and ‘selling our content’ (P8). For other participants, internationalisation and global citizenship were buzz words, described as ‘flavoursome’ (P1), a ‘fashionable term’ (P4) and a ‘throw out phrase’ (P1). ‘Uncertainty’ and ‘ambiguity’ were also terms used by multiple participants suggesting it to be ‘vague’ in its intentions in occupational therapy and consequently, of questionable benefit to the profession and in the curriculum.

Teaching practices in occupational therapy were not considered relevant to internationalisation of the curriculum, with an experienced academic stating it is ‘not well defined in my Uni nor am I aware of it as someone designing curriculum’ (P2). Another participant wondered whether the aim was to establish ‘one curriculum internationally’ (P3), while another reflected, ‘what does internationalisation of the curriculum even mean? Does that mean you want students to be able to work anywhere in the world?’ (P1). Similarly, for two academics, this was the first time they had encountered global citizenship with one stating ‘I don't think anything that I would've done would've constituted [global citizenship] because that's not a term that I use’ (P6). In this category, institutionally imposed global citizenship and internationalisation of the curriculum were considered to have little relevance to occupational therapy education. Perspectives shared by participants therefore appeared to be on a continuum dependent on the context of global citizenship with elements of global citizenship embedded in curricula when not linked to the perception that it is institutionally imposed.

#### 4.2.2 Category 2: Locally mandated and important

Internationalisation of the curriculum and global citizenship more closely aligned with the need for students' awareness of local cultural differences and preparation for practice in the *local context*. One participant stated, ‘[students] can look overseas and go “Oh there's all these issues” but a lot of [students] aren't aware of what's happening in Alice Springs or in the Northern Territory’ (P16). When asked about student preparation in relation to culture, responses were described primarily with reference to Aboriginal and Torres Strait Islander Peoples with little discussion about other cultural factors such as ethnicity, gender, or sexual orientation. As one participant reflected ‘I have a lot of Indigenous content on purpose because I think … in Australia one of the things that we need to deal with first … is cultural differences with the First Nations People’ (P2). Teaching about Aboriginal and Torres Strait Islander knowledges was not only a priority but was considered the best way to prepare culturally respectful and responsive graduates. Teaching strategies included a ‘buy‐in strategy’ where specialist teachers were brought in to teach Indigenous content, which participants felt reflected some element of internationalisation of the curriculum. This was seen as the best way to ensure occupational therapy accreditation standards and education related to diversity were met.

National accreditation and competency standards (Occupational Therapy Board of Australia, 2018) and the requirement in Australia to map occupational therapy curriculum to the Aboriginal and Torres Strait Islander Health Curriculum Framework (Department of Health, 2014) were strong drivers for guiding curriculum development. This may have in part influenced an audit culture surrounding program accreditation within this category.

Accreditation has single‐handedly transformed occupational therapy education of including Aboriginal and Torres Strait Islander curriculum … by having it as an accreditation standard, [it's] making us do it … that has had a massive impact on how we resource that aspect of our curriculum [and] that definitely has influenced what we do. (P11)

Despite the requirements to conform to national accreditation and competency standards (OTC, 2024) participants felt ill‐equipped and unsure about how to internationalise their curriculum further with an already ‘crowded curriculum’ (P8). Educators described discomfort in the classroom as another key reason why they were resistant to do more than what was required for accreditation and felt addressing local diversity was sufficient.

#### 4.2.3 Category 3: Values driven and desirable

Several participants in Category 3 viewed the inclusion of multiple cultural perspectives in curricula as essential for preparing students for professional practice. One participant felt ‘the Australian way of thinking and being and doing’ (P14) limited readiness for working with diverse cultures beyond Aboriginal and Torres Strait Islander Peoples. Students were seen as needing exposure to varied socio‐economic backgrounds, cultural roles, gender, and political views, important for understanding the impact this has on occupational therapy practice. Classroom diversity was seen as a valuable resource for exploring culture's influence on occupational participation. The ability to create ‘culturally safe learning spaces’ (P14) was described as an increasingly important teaching skill. Other participants drew on professional experiences in their teaching to help broaden students understanding of the world and its people with one participant sharing.

I worked with Welsh travelling communities … in rural Wales … some [families] lived in caravans, so trying to get access to a caravan for a child with a disability is really difficult. If you can imagine how accessible the bathroom is in a caravan. (P6)

Without the inclusion of other cultural factors, one participant felt students' risk being ‘incredibly unsophisticated in their view of the world and other people’ (P4). Another participant described being questioned by a student as to why sexual preference was included in a case study. Participants felt it was therefore essential for students to ‘understand that occupations are culturally shaped and framed’ (P9), valuing global citizenship because it enabled students to look at their ‘own unconscious biases and how [they] can actually misinterpret what's going on because of [their] own upbringing, culture [and] values’ (P2). The inclusion of ‘multiple’ cultural factors was considered the best approach to preparing students for professional practice. However, participants felt unskilled in developing an internationalised curriculum beyond informal classroom discussions: ‘We've been talking about a curriculum refresh; we were just not quite brave enough to take that next step … we didn't feel like we had the skills’ (P13). The lack of a shared goal was also described as a barrier with another participant stating ‘I am only one part of an academic team, there are other people that are subject co‐coordinators and have their own agendas’ (P14).

#### 4.2.4 Category 4: Professionally essential and aspirational

Internationalisation of the curriculum and global citizenship were perceived as *essential* because occupational therapy is a ‘global’ profession. Global citizenship was viewed as critical for becoming an effective, ‘global professional’, a privileged position with the potential to have a positive impact on the world. Being a global professional, required students to understand ‘where the profession sits in the world’ (P15) and that they are ‘part of [a] bigger global community of occupational therapists’ (P15).

Acknowledgement of diversity, power, and privilege were seen as important to include in occupational therapy education. Internationalisation of the curriculum and global citizenship were perceived as useful concepts because it prompted reflection on ‘all of those really big conflicts around, [how do] my actions, and the occupations that I engage with, influence [those] and how are they influenced by [them]’ (P18). A sound understanding of human rights was considered a prerequisite for effective practice with one participant stating students need to know ‘what poverty means, of what it is to not have human rights, or not have a roof on your head’ (P1) to prepare them for the diversity they will inevitably face in practice. Another participant suggested an ‘understanding of international politics and where people are coming from and going [to] particularly with the refugee populations’ (P16) was essential to include in curricula.

Global citizenship was associated with ‘addressing injustices that are in society … and helping to support people to address those injustices’ (P14). Teaching priorities related to ‘higher‐level contextual factors, political systems and making a more fair and just world’ (P8). Participants described this as not only ‘the goal for me in terms of occupational therapy’ (P8) but also ‘some of the most important parts of the program’ (P14). Aspirations for educators to commit to ‘social responsibility’ were highlighted with one participant stating ‘our humanity connects us’ and using personal agency ‘to do good, to improve things … [to] challenge those structures that oppress people … in an ethical way, a responsible way’ (P14) is essential. Personal values grounded in equity and justice and life experience were evidenced in participants' sense of professional responsibility. For some participants the interview served as an opportunity to critically reflect on self‐posed questions such as ‘how [are we] supporting our graduates to be best placed to practice and make a difference?’ (P18).

A transformation to curriculum was one suggestion, with internationalisation of the curriculum seen as the key vehicle for preparing students to think more critically and politically about justice and human rights. Focusing on the structure of curriculum, one participant suggested

students need repeated, scaffolded opportunities to talk about culture, to understand diversity, to understand themselves … it should be vertically and horizontally embedded (P14).

Some recognised the aspiration of transforming curricula but noted there were perceived challenges with curriculum change and felt that advocacy for change was essential to address the increasing complexity in the world. One participant suggested ‘global occupational therapy should be the norm in curriculum’ (P4) while another stated ‘it's really important for [students and educators] to understand that this is a living, breathing profession and s as things emerge and develop the profession's got to be responsive’ (P15). The importance of becoming a global professional and citizen was thought of as being ‘part of life and education’ (P12).

## 5 OUTCOME SPACE

The phenomenographic method of analysis culminates in an overarching ‘outcome space’ which highlights variations in the categories described earlier (Akerlind, 2017). The outcome space presents an in‐depth analysis of the descriptions through the external horizon (curricula) and the internal horizon which is a culmination of participant's awareness of global citizenship and internationalisation of the curriculum and the dimensions/factors that contribute to the variation identified between categories.

### 5.1 External horizon

We applied Leask's (2010, 2012) *internationalisation of the curriculum framework* to the categories described to identify whether participants spoke of global citizenship in the formal (planned), informal (extracurricular activities), and/or hidden (classroom discussions) curriculum (described in the outcome space as the ‘external horizon’). Understanding where global citizenship is being placed within curricula enables a more in‐depth analysis of the factors that contribute to the variations of participants' perceptions/experiences of global citizenship. For example, some factors contributing to the variations between categories included teaching practices, the drivers towards participants' perceptions, and the perceived relationship between global citizenship and internationalisation of the curriculum.

### 5.2 Internal horizon

Within the outcome space, the internal horizon highlights the main focus of awareness of participants' descriptions within each category. It also presents what was missing from participants' awareness in relation to the framework for internationalising curriculum. The internal horizon (main focus) in Category 1 highlights global citizenship and internationalisation of the curriculum as institutionally imposed whereas in Category 4, the internal horizon (main focus of awareness) was global citizenship as professionally essential and aspirational (see Table 2). Similarly, by applying Leask's framework, we were able to identify what dimensions of the framework (i.e., international, intercultural, and global dimensions) and what contexts (i.e., institutional, local, national, regional, and global) were missing from participants' awareness (i.e., not described in their responses to interview questions) in each of the four categories.

**TABLE 2:** Outcome space.

Structural								
	Category of description	External horizon	Internal horizon		Dimensions of variation			
			Focus of awareness	Missing from the focus of awareness	Driver for enacting internationalisation of curriculum for global citizenship	Examples of teaching approaches	Perceived relationship of internationalisation of curriculum for global citizenship and teaching	Soft/critical global citizenship
Referential	1. Institutionally imposed and irrelevant	No curriculum	Irrelevant, added‐on concept imposed by the institutional context.	International, intercultural, and global dimensions; local, national, regional, and global contexts.	Institutional, commercialised agenda, imposed by higher education.	International student mobility, mainly recruitment of international students.	An added component to student learning, with little relevance to occupational therapy curriculum.	Neither.
	2. Locally mandated and important	Formal curriculum	Relevant to local context, respecting cultural differences; pragmatism.	International and global dimension, international and global contexts, and educators as active learners.	Occupational therapy accreditation standards to renew curriculum.	Case studies, lectures, cultural immersion days, role‐emerging placements, and exams.	A relevant component to student learning of culturally safe practice with aboriginal and/or Torres Strait islander peoples.	Soft approach focused on cultural inclusiveness.
	3. Values driven and desirable	Formal and hidden curriculum	Modelling global citizenship by valuing diversity in the classroom and using experience to prepare students for practice.	Global dimensions, global context, and educators as active agents of change.	Personal and professional participant experiences but resistance to curriculum change due to discipline agendas.	Informal discussions, collaborative learning, peer teaching on international perspectives, and international guest speakers.	An integrated component to student learning to prepare students for local and international professional practice.	Soft approach focused on morally doing good with regards to multiple diverse factors.
	4. Professionally essential and aspirational	Formal, informal, and hidden curriculum	Essential for becoming a global professional and citizen through critical reflection, thinking politically and enacting social responsibility; challenging existing curriculum.	Learning outcomes and assessments.	Participant values and professional responsibilities, aspiring to revolutionise curriculum.	Critical reflections, video diaries, students attending/presenting at conferences, international online collaborative learning, simulations (e.g. refugees), global politics modules, human rights and social ethics literature, bicultural advisory panels, and leadership and mentoring.	An inherent and scaffolded component to student learning to develop students as global professionals and global citizens.	Critical approach focused on self‐reflection and action related to political justice, human rights, and social responsibility.

The dimensions of variation between each category are also presented in the outcome space. Using Canadian Indigenous educator and scholar Andreotti's (2006) *soft*–critical framework for global citizenship, we highlighted the extent of participants' understanding about global citizenship as either soft or critical. Additionally, we identified the drivers for the perceptions of global citizenship and internationalisation of the curriculum in occupational therapy in each category to ensure a robust overview of how each category represents a more advanced understanding of global citizenship in occupational therapy education. Descriptions therefore represent the level of awareness about global citizenship and internationalisation of the curriculum in occupational therapy education within that category. For example, in Category 1, there was no approach to global citizenship identified. In contrast, in Categories 2 and 3, a soft approach was identified whereby the focus of participants' descriptions was on cultural inclusiveness. Category 4, which represents the most advanced perception of global citizenship in occupational therapy education, was the only category to have a critical approach to global citizenship identified and described by participants. In this category, perceptions of global citizenship focused on self‐reflection and action related to political justice, human rights, and social responsibility.

The external and internal horizon and a summary of the dimensions contributing to variations between the four categories are presented in Table 2.

## 6 DISCUSSION

The outcome space highlights a range of factors and drivers that led to the perceptions participants had about the importance of global citizenship in occupational therapy education. *Drivers* influence and impact what disciplinary knowledge is considered important in curriculum. Using the viewpoints of educators expressed in Category 1, global citizenship was considered an added‐on concept in occupational therapy education with little relevance, imposed by the institution. Institutional policymakers often describe at a macro level what the vision, values, and desired graduate attributes of a particular institution are (e.g., graduate employability skills; Borkovic et al., 2020). However, it is likely that institutional policymakers are providing minimal guidance to educators on how to meaningfully embed graduate attributes in discipline specific curriculum such as global citizenship. In Category 1, teaching practice was identified as student mobility or recruiting international students, and learning was linked with the idea of developing intercultural competence (Fortune et al., 2019). While educators may have been embedding elements of global citizenship through these teaching practices, this approach is least likely to prepare students for working with culturally diverse populations because not all students engage in mobility through study or work integrated learning (Elliot, 2015).

The Category 2 driver of thought and action was accreditation. *Accreditation bodies* appeared far more influential in occupational therapy education than the institution. Accreditation bodies in Australia have the power to dictate elements of the curriculum through auditing and approval processes (Australian Health Practitioner Regulation Agency [AHPRA], 2024). Conceptualisations in this category related mostly to meeting accreditation requirements such as cultural safety and responsiveness when working with Aboriginal and Torres Strait Islander Peoples. Cultural safety requires graduates to analyse power imbalances, discrimination, and the ongoing effects of colonisation (Beagan, 2015). Cultural humility requires flexibility, openness, and critical reflexivity to redress power imbalances between clients and therapists and the hierarchical nature of cultural difference (Beagan, 2015). Cultural responsiveness requires commitment to self‐reflection on social and cultural factors when working with people of diverse cultural backgrounds (Occupational Therapy Board of Australia [OTBA], 2018). In an increasingly globalised world, a larger set of graduate attributes including political awareness, social responsibility, and global citizenship attributes are required of occupational therapists that extend beyond safety, responsiveness, and humility. These broader graduate attributes could be considered by the OTBA in the next revision to the Occupational Therapy Competency Standards, last published in 2018 (OTBA, 2018) and are in keeping with the educational position statement published in 2021 by the Word Federation of Occupational Therapists.

Critical reflections on social positioning and personal and professional actions to decolonise are called for in occupational therapy literature (Emery‐Whittington, 2025). Perceptions of educators that the focus should be on decolonising curricula ‘locally’ are of importance. Broadening the perspective of culture towards intersectionality, where teaching teams are encouraged to authentically share collective experiences with students, may inspire critical reflections and higher level learning that enables increased skill and knowledge and the inclusion of other cultural factors deemed important such as ethnicity, gender, and class (Hammell, 2013). This process is influential in helping teams to feel, imagine, and relate differently (Stein & Andreotti, 2021); reduces misconceptions about global citizenship (Fortune et al., 2021); and can be transformative (Kirk et al., 2018) in how educators can better prepare students for professional practice.

In Category 3, educators used professional experiences in classroom discussions as a resource for students to learn about the influence and impact of culture on occupational therapy practice both locally and internationally. Within this category, the viewpoint that intersectionality was an important part of student learning was highlighted. Participants with this view have been described as *thought leaders* (Gutman, 2025). Thought leaders are inspirational, passionate education, and research advocates, capable of evoking deep and critical thought in a professional community. Thought leaders have the power to influence what occupational therapy educators do and are instrumental to (the process of) graduates developing a stronger sense of self and professional identity. Participants were not asked about intersectionality in the curriculum specifically, but rather the broad term ‘culture’ was used, allowing participants to respond with cultural factors that came to mind. Topics such as ethnicity, gender, and sexuality that occur organically were identified in the hidden and often implicit curriculum by only some participants (Hooper et al., 2018); however, they fall short in preparing graduates for professional practice in occupational therapy without scaffolding in the formal and informal curricula too (Leask, 2012).

A learner‐centred approach that enables diverse ways of thinking and acting is important (Leask, 2010, 2012). In their teaching practices, educators should role‐model critical reflection on their social and political identities and the influence these identities have on their understanding of occupational therapy, challenging dominant paradigms of occupational therapy education (Emery‐Whittington, 2025). Engaging in simulation and/or real‐life community engagement in a range of culturally diverse contexts enables students to practice skills, acquire new insights, think differently about occupational therapy practice, and build their knowledge base on social and political responsibilities and practices (Lopes et al., 2025; Sakellariou & Pollard, 2013).

The vision and values of the occupational therapy profession strongly influence its aspirations in education, with values‐based learning and global citizenship increasingly emphasised alongside decoloniality and culturally relevant theory and practice (Emery‐Whittington, 2025; OTC, 2024; Rudman et al., 2021; WFOT, 2021). The values held by the profession, therefore, such as those perceptions shared in Category 4, will most strongly influence what is included in occupational therapy education over time. Disciplinary advocates are needed within education programs to draw on emerging knowledge paradigms that challenge traditional occupational therapy paradigms despite the possibility of discomfort in curriculum change, as highlighted in participant perceptions included within Category 4 of the outcome space. Disciplinary advocates are committed to developing their own sense of global citizenship and identity and bring new understandings into course content and teaching practices (Cochrane et al., 2017). It is, therefore, the responsibility of educators to help challenge current educational approaches, emphasising the importance of learning about both the local and global context and the injustices restricting all people from their rights to engage in occupation.

Experiential and transformative learning are the most powerful learning experiences for professional development, aligning with the aspirations outlined in Category 4 of the outcome space. Transformative learning in the occupational therapy literature is understood as preparing students to become ‘more inclusive, reflective, emotionally flexible and refined’ (Crawford et al., 2017, p. 131) in their occupational therapy practice. Students engaging in transformational learning experiences in which they often experience discomfort or challenge are better prepared to enact social change and are more knowledgeable in the areas of human rights, equity, access, and diversity and inclusion. Encouraging discussion and debate of social and political issues through a rights‐based lens fosters global citizenship and deepens learning (Kenna, 2017). Topics such as refugeeism, unemployment, food insecurity, and systemic oppression such as policy and governance change (Frank, 2012) can enhance students' ability to address social barriers to occupational engagement and participation for individuals, communities, and populations (Gutman, 2025).

Political and social responsibilities are increasingly called for in occupational therapy whereby graduates are politically aware and act ethically to promote and enable justice (Dos Santos & Frank, 2025; Gutman, 2025; Lopes et al., 2025; Sakellariou & Pollard, 2013). Attributes associated with being politically aware enables students and graduates to identify social and political actors and stakeholders, their motives and desires. Attributes associated with acting ethically enables students and graduates to negotiate the positions people hold in society which enable or restrict participation. Some examples in curriculum might be learning activities and assessments that require students to take on advocacy roles and deliver a pitch to a governing body. Students could consider who the key stakeholders are (governance, community, individual, etc.), whose goals are being prioritised (e.g., efficiency verse inclusion or cost saving verse community needs), and making the hidden visible by calling out injustice and working collaboratively to find meaningful resolutions (e.g., advocating for changes to funding and eligibility criteria or institutional routines that promote inclusion). Further, global citizenship capabilities require graduates to address if and how they might contribute to power imbalance/inequity (Andreotti, 2006, 2021). It is therefore important that students experience learning opportunities within the formal, informal, and hidden curriculum (Leask, 2012) that explore the occupational restrictions and injustices that are increasingly apparent in global occupational therapy practice (e.g., injustice related to gender, class, diversity, and ability) and that this learning is assessed throughout a program of study.

## 7 CONCLUSION

Preparation for professional practice within a culturally diverse Australia is important. Without appropriate preparation, occupational therapy education risks producing graduates who are underskilled and unprepared to serve a culturally diverse population, further risking the perpetuation of power imbalance. It is therefore important to critically examine the various ways Australian occupational therapy educators prepare graduates with the skills and knowledge required to serve the local, diverse, and multicultural population within Australia. Developing graduates as global citizens equips them to work comfortably in a complex and changing society, capable of addressing adversity and injustice and may contribute to building a stronger more connected and more resilient occupational therapy workforce.

## AUTHOR CONTRIBUTIONS

All authors meet the criteria for authorship.

## CONFLICT OF INTEREST STATEMENT

The authors declare no conflicts of interest.

## Data Availability

The data that support the findings of this study are available from the corresponding author upon reasonable request.
